# Combining bulk and single-cell RNA-sequencing data to reveal gene expression pattern of chondrocytes in the osteoarthritic knee

**DOI:** 10.1080/21655979.2021.1903207

**Published:** 2021-03-22

**Authors:** Xiaoyu Li, Zheting Liao, Zhonghao Deng, Nachun Chen, Liang Zhao

**Affiliations:** a Department of Orthopaedics, Nanfang Hospital, Southern Medical University, Guangzhou, China; b Key Laboratory of Bone and Cartilage Regeneration Medicine, Nanfang Hospital, Southern Medical University, Guangzhou, China; c Department of Orthopaedics, Shunde Hospital, Southern Medical University, Foshan, China

**Keywords:** Osteoarthritis, chondrocyte, bulk RNA sequencing, single-cell RNA sequencing, bioinformatics

## Abstract

Osteoarthritis (OA) occurs mostly in the knees, hips, finger interphalangeal joints, and spinal facet joints, and is characterized by cartilage degeneration. The existing bulk RNA sequencing (bulk RNA-seq) and single-cell sequencing (scRNA-seq) data for chondrocytes in the osteoarthritic knee joint provide the expression profiles of entire cell populations and individual cells, respectively. Here, we aimed to analyze these two types of sequencing data in order to obtain a more comprehensive understanding of OA. We compared the analysis results of bulk RNA-seq and scRNA-seq from the dataset GSE114007 and the dataset GSE104782, respectively, and identified the differentially expressed genes (DEGs). Then, we tried to find the key The transcription factor is a more fomal term (TFs) and long non-coding RNA (lncRNA) regulation. We highlighted 271 genes that were simultaneously suggested by these two types of data and provided their possible expression pattern in OA. Among the 271 genes, we identified 14 TFs, and TWIST2, MYBL2, RELA, JUN, KLF4, and PTTG1 could be the key TFs for the 271 genes. We also found that 8 lncRNAs among the 271 genes and the lncRNA regulation between CYTOR and NRP1 could contribute to the pain and vascularization of cartilage in the osteoarthritic knee. In short, our research combined the analysis results of bulk RNA-seq and scRNA-seq data for OA chondrocytes, which will contribute to further elucidation of the molecular mechanisms of OA pathogenesis.

**Figure:**
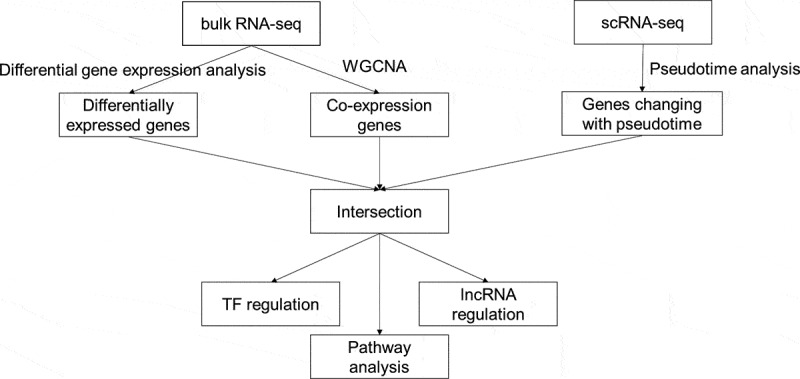

## 1. Introduction

Osteoarthritis (OA) is the most common form of arthritis affecting the joints, including knees, hips, finger interphalangeal joints, and spinal facet joints. And it usually occurs after middle age, causing joint pain and restricted activity [1]. OA is the leading cause of lower limb disability in the elderly. Estimated 240 million people worldwide suffer from OA [2], with symptomatic OA found in 9.6% of men and 18.0% of women over 60 years old (World Health Organization, n.d.). Pathologically, OA is a degenerative disease due to the cartilage deterioration that occurs. Cartilage, which is the main lesion site, has been the focus of OA research.

Sequencing technology can reveal intracellular transcription and further assist in inferring signaling pathways. Bulk RNA sequencing (bulk RNA-seq) and single-cell RNA sequencing (scRNA-seq) are currently used in mainstream sequencing technologies. They reflect cell transcription at different levels. Bulk RNA-seq provides a tissue-wide transcription landscape. It enables deep investigation into the transcriptome, but it may obscure critical differences between individual cells. The emerging scRNA-seq reveals the expression profiles of individual cells [3]. Due to the small amount of material that is analyzed, it is not possible to obtain information as deep as that obtained by bulk RNA-seq, although patterns of gene expression can be identified through gene clustering analysis [3,4].

The sequencing data for human chondrocytes in the osteoarthritic knee exist for these two types of sequencing. Studies have examined bulk RNA-seq sampled from healthy and end-stage osteoarthritic cartilage. Chen et al. [5] used RNA-Seq to screen the differentially expressed genes (DEGs) in chondrocytes of normal adults and patients with knee OA. Fisch et al. [6] collected knee cartilage tissue from 5 females and 13 males (aged 18–61 years, mean 38) normal human, and 12 females and 8 males (aged 52–82 years old, mean 66) patients with OA. RAN-seq revealed 1332 DEGs in OA and non-OA samples. The only scRNA-seq study involved 1464 labeling chondrocytes that were obtained from 10 end-stage osteoarthritic cartilages with different pathological stages of cartilage lesions [7]. Thus, the bulk RNA-seq studies could reflect the overall gene expression differences between OA and normal samples, and the scRNA-seq study could simulate the dynamic gene expression pattern of OA progression. Previous bulk RNA-seq studies focused on differentially expressed genes (DEGs) between OA and normal samples or co-expressed genes under OA or normal conditions, and revealed several dysregulated transcription factors (TFs) [6,8], microRNAs (miRNAs) [5,9], long non-coding RNAs (lncRNAs) [10–12], and circular RNAs (circRNAs) [13]. The scRNA-seq study focused on identifying different cell clusters of OA chondrocytes and, to some extent, explored the expression of transcription factors for early-stage and late-stage OA [7].

To identify genes that play an important role in the pathogenesis of OA and their expression pattern, we compared the genes suggested by these two types of data from the dataset GSE114007 and the dataset GSE104782, respectively, of the knee OA samples, using their own unique analytical methods. Based on the genes that were simultaneously suggested by these two types of data, we further explored the key transcriptional regulators and long non-coding RNA (lncRNA) regulation among them. Our research may provide some enlightenment for the understanding and treatment of OA.

## 2. Materials and methods

### 2.1 Data collection and preprocessing.

The Gene Expression Omnibus (GEO) database was searched using the keywords ‘Osteoarthritis,’ ‘cartilage,’ ‘chondrocyte,’ ‘knee joint,’ and ‘human.’

Samples from the dataset GSE114007 [6] were separately sequenced on two platforms, Illumina HiSeq 2000 (GEO accession number: GPL11154) and Illumina NextSeq 500 (GEO accession number: GPL18573), using an RNA-seq technique. There were 38 samples, 18 of which were derived from tissue banks and were normal knee cartilage tissues without joint disease or trauma history, and 20 of which were OA-affected tissues harvested from knee arthroplasty. The normal tissues came from 5 women and 13 men (18–61 years of age, mean 36.6). The OA tissues were derived from 12 women and 8 men (51–82 years of age, mean 66.2).

The dataset GSE104782 [7] followed a modified single-cell tagged reverse transcription (STRT) protocol to generate sequencing libraries and was sequenced on an Illumina HiSeq 4000 platform. Knee cartilage was procured from 10 patients undergoing knee replacement surgery. Distinct areas of cartilage were divided into five different stages, S0 to S4, corresponding to the five grades of ICRS cartilage lesion classification system ranging from ‘normal’ to ‘severely abnormal’. For each patient staging area, a sample of 32 cells was removed, and a total of 1600 chondrocytes were obtained. After quality control, 1464 chondrocytes were suitable for subsequent analysis. The samples were from seven women and three men (60–77 years of age, mean 66.5).

Because the datasets were from different time periods, the gene symbols may be inconsistent. We corrected the gene symbols based on the online tool of official gene symbols (HUGO Gene Nomenclature Committee, n.d.). The procedure was as follows: previous or alias symbols were replaced with approved ones; if more than one approved symbol was matched with one symbol, only the first one was selected; the withdrawn gene symbols were removed, and the median expression value of the repeated probes was taken.

### 2.2 Differential gene expression analysis

R package DESeq2 v1.28.1 [14] was utilized to perform differential gene expression analysis of bulk RNA-seq data comparing osteoarthritic samples with normal samples, taking into account the batch effect of different sequencing platforms. We use the default Wald test for differential expression analysis. The method used for adjusting p-values was Benjamini–Hochberg method. Genes with adjusted p value less than 0.05 and absolute value of fold change greater than 2 were considered to be DEGs. The logarithmic transformed data were calculated using DESeq2 and the batch effect of sequencing platform was removed using the R package limma v3.44.3. After batch effect correction, the data were used for principal component analysis (PCA) and weighted correlation network analysis (WGCNA).

### 2.3 WGCNA

R package WGCNA v 1.69 [15] was used to identify the gene modules most relevant to the OA. The network adjacency was calculated by squared Euclidean distance. A sample would be discarded if its standardized connectivity was less than −2.5. A signed network was built, and the correlation analysis employed bi-weight mid-correlation. The soft threshold enabled the scale-free topology fit index to reach 0.85.

### 2.4 Pseudotime analysis

The R package monocle v2.16.0 [16–18] was applied for pseudotime analysis, also known as single-cell trajectory analysis. To obtain the genes required for calculating pseudotime, differential gene expression analysis was performed among chondrocytes from different pathological stages. After calculating the pseudotime, the differential gene expression analysis was repeated to determine the genes that changed as a function of pseudotime. The cell state containing the greatest number of S0-stage cells was considered as the root state. The threshold of the q value for multiple testing involved in the selection of DEGs was 0.01.

***2.5 Gene Ontology (GO) and Kyoto Encyclopedia of Genes and Genomes (KEGG) pathway enrichment analysis***

We utilized clusterProfiler [19] to find out the GO/KEGG pathways in which the given gene sets are enriched. The threshold of p value was 0.05. The Benjamini-Hochbergch procedure was used to adjust the p value. The cutoff value of q value was 0.2.

### 2.6 Integrated analysis

We intersected the genes obtained from differential gene expression analysis, WGCNA, and pseudotime analysis. The intersection of upregulated DEGs, the gene module having the strongest positive correlation with OA, and genes that changed as a function of pseudotime were defined as upregulated genes of interest. In addition, the intersection of downregulated DEGs, the gene module having the strongest negative correlation with OA, and genes that changed as a function of pseudotime were defined as downregulated genes of interest. TFs were identified based on the Human Transcription Factor Database (HumanTFDB) v3.0 [20]. Key transcriptional regulators for genes of interest were found via Transcriptional Regulatory Relationships Unraveled by Sentence-based Text mining (TRRUST) v2 [21]. The threshold of the q value for key regulators was 0.05. LncRNAs were recognized by the lncRNA annotation of gene symbol in HGNC BioMart (HUGO Gene Nomenclature Committee, n.d.) database. LncTarD [22], a database for experimentally supported functional lncRNA–target regulators in human diseases, was searched for potential lncRNA regulation within genes of interest. Cytoscape v 3.7.2 was used to visualize the regulatory relationship.

## 3. Results

### 3.1 Differential gene expression analysis.

**Figure 1.**
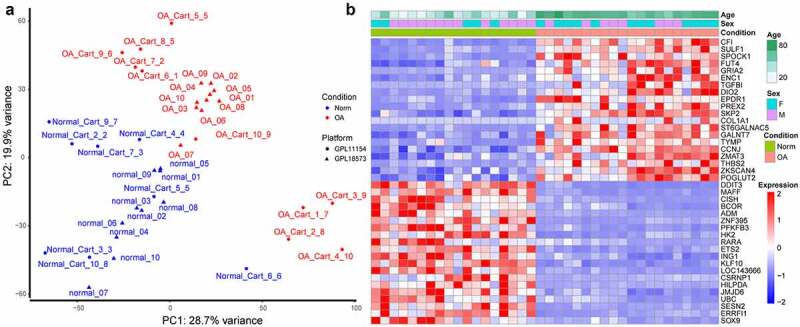
Sample clustering and differentially expressed genes (DEGs) between osteoarthritic (OA) and normal samples. (a) principal component analysis (PCA) plot of samples after removing batch effect. PC1: the first principal component; PC2: the second principal component. (b) heat map for DEGs. The top 20 upregulated or downregulated DEGs ranked by adjusted p values are displayed

Based on the PCA results, there was a certain degree of discrimination between normal and OA samples (Figure 1a). The samples roughly clustered according to the disease condition. We obtained 1375 upregulated DEGs and 1026 downregulated DEGs. Ordered by adjusted p value, the top five upregulated or downregulated DEGs were CFI, SULF1, SPOCK1, FUT4, GRIA2, and DDIT3, MAFF, CISH, BCOR, ADM, respectively (Figure 1b). Several pathways were suggested by GO and KEGG pathway analysis of these DEGs (Supplementary Figure S1). Among the GO pathways, the most significant upregulated pathways in OA tissues were related to extracellular matrix, and the most significant downregulated GO pathways involved response to nutrition and hormones. The most significant upregulated KEGG pathways were focal adhesion, cell adhesion molecules (CAMs), phagosome, and complement and coagulation cascade. The most significant downregulated KEGG pathways include MAPK, FoxO, hypoxia-inducible factor 1 (HIF-1) and tumor necrosis factor (TNF) signaling pathway, and circadian rhythm.

### 3.2 WGCNA

**Figure 2.**
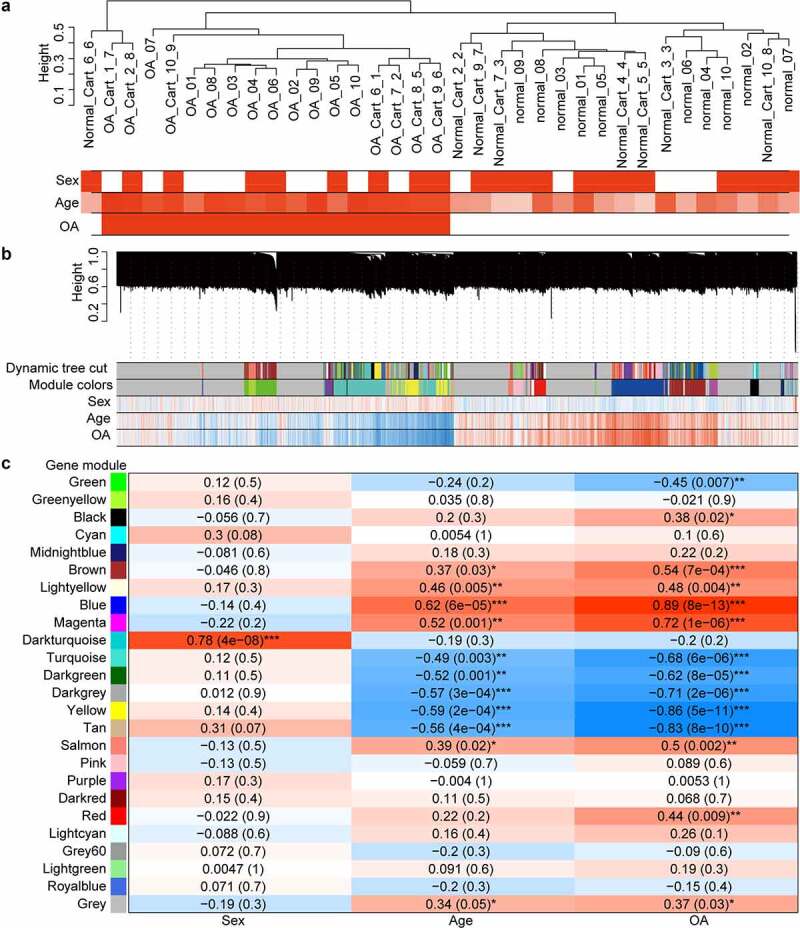
Cluster dendrograms and correlation heat maps. (a) cluster dendrogram of samples and trait heat map. White denotes low and red denotes high. In terms of the sex trait, 0 indicates female, and 1 indicates male. For the OA trait, 0 denotes normal and 1 denotes OA. (b) Cluster dendrogram of genes and gene-trait correlation heat map. The different colors on the left side of the heat map represent different gene co-expression module. Blue denotes low and red denotes high for the correlation heat map. (c) Module-trait correlation heat map. The numbers outside the parentheses represent the correlation coefficient, and the numbers in parentheses represent the p-value. *: p < 0.05; **: p < 0.01; ***: p < 0.001

The samples clustered well according to their OA or normal phenotype (Figure 2a). When a gene was positively correlated with OA, it also had a strong positive correlation with age and a weak negative correlation with being male (Figure 2b). The modules having the strongest positive and negative correlation with OA (Figure 2c) contained 1240 and 731 genes, respectively. The hub genes of the modules having the strongest positive and negative correlation with OA were SLC35B4 and MAFF, respectively. The genes of module having the strongest positive correlation with OA were mainly enriched in the extracellular matrix-related pathways and the enrichment results of the module with the strongest negative correlation to the OA showed FoxO and HIF-1 signaling pathway again (Supplementary Figure S2).

### 3.3 Pseudotime analysis

**Figure 3.**
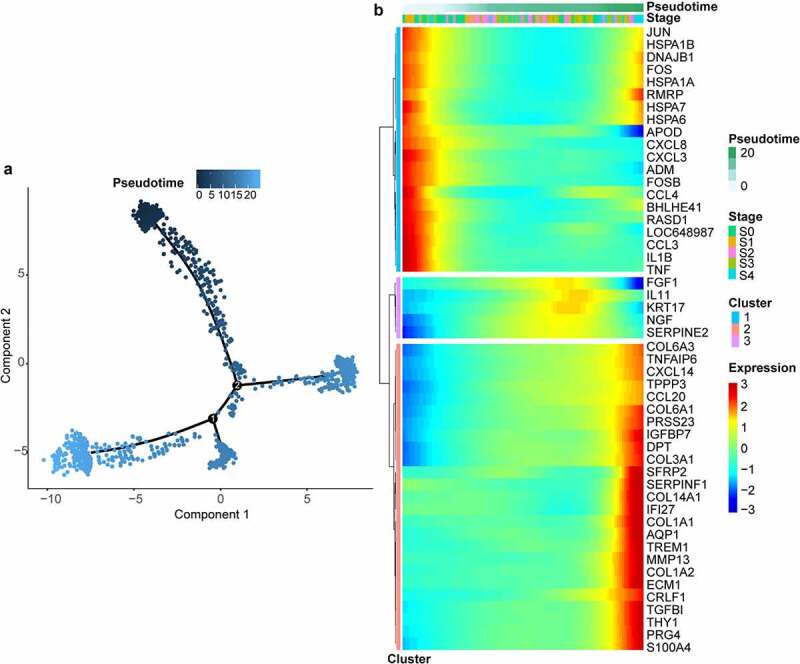
Gene expression along pseudotime and enriched pathways. (a) cell trajectories colored by pseudotime. (b) modules of genes covarying across pseudotime

We constructed a pseudotime model to reflect the dynamic gene expression change between chondrocytes from different pathological stages of cartilage under microscope. There was a trend that the more severe the pathological stage of cartilage from which a chondrocyte came, the pseudotime allocated to the chondrocyte would be higher. We obtained 3139 genes that changed as a function of pseudotime. As the heat map of cell sorting by pseudotime indicated (Figure 3), CCL3, CXCL8, and IL1B were mainly upregulated at the start of the pseudotime, COL1A1, COL1A2, and PRG4 were upregulated near the end, and FGF1, KRT17, and NGF were upregulated during the middle.

### 3.4 Integrated analysis

**Figure 4.**
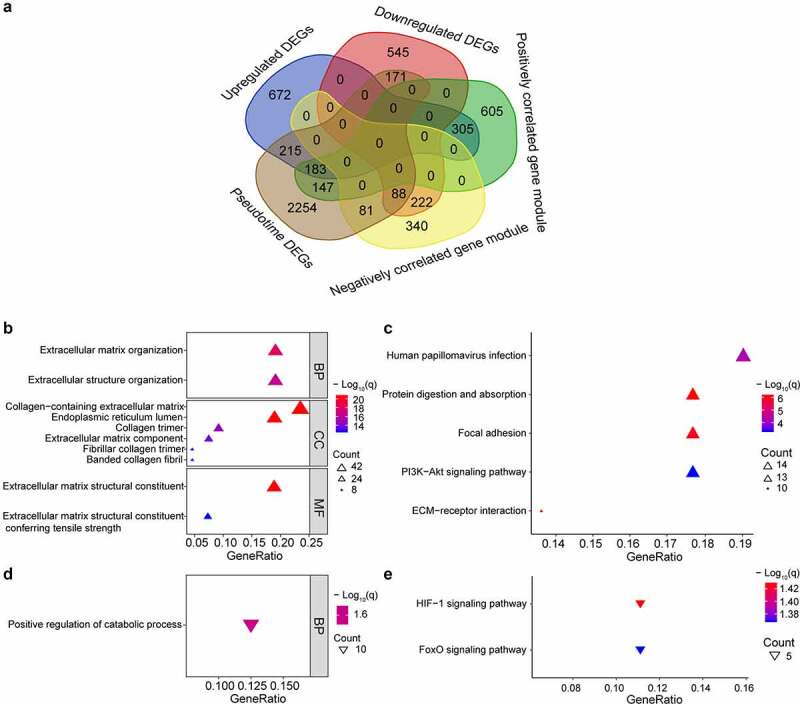
Intersection of genes obtained from different methods and enriched pathways. (a) intersection of differentially expressed genes (DEGs), gene modules having the strongest correlation with OA, and genes changing as a function of pseudotime. Positively correlated gene module: the gene module exhibiting the strongest positive correlation with OA. Negatively correlated gene module: the gene module exhibiting the strongest negative correlation with OA. Pseudotime DEGs: genes changing as a function of pseudotime. (b) top 10 upregulated gene ontology (GO) pathways ranked by q value. BP: biological process; CC: cellular component; MF: molecular function. (c) significant upregulated kyoto encyclopedia of genes and genomes (KEGG) pathways. (d)significant downregulated GO pathways. (e) significant downregulated KEGG pathways

There were 183 upregulated genes of interest at the intersection of upregulated DEGs, genes within the gene module having the strongest positive correlation with OA and genes changing as a function of pseudotime. And there were and 88 downregulated genes of interest at the intersection of downregulated DEGs, genes within the gene module having the strongest negative correlation with OA and genes changing as a function of pseudotime (Figure 4a, Supplementary Table S1). Many of the upregulated genes were upregulated near the end of the pseudotime, and many of the downregulated genes were upregulated near the start of pseudotime (Supplementary Figure S3). The upregulated genes were enriched in the extracellular matrix-related pathways and the downregulated genes were enriched in the HIF-1 and FoxO signaling pathways (Figure 4b-E).

**Figure 5.**
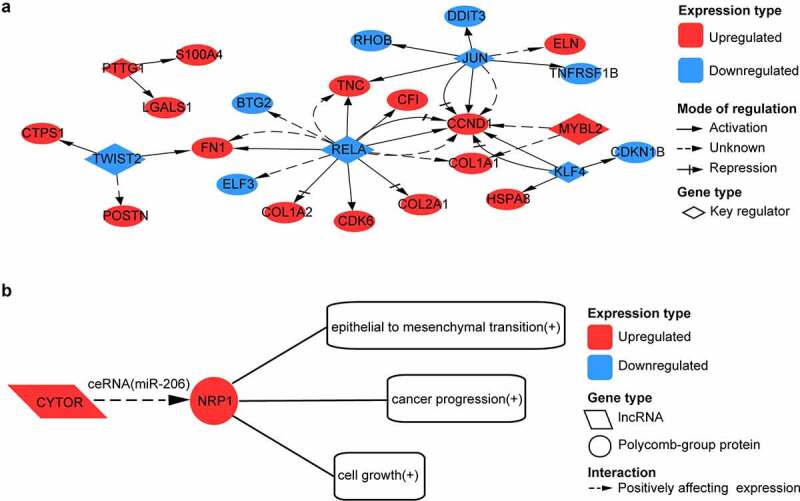
Analysis of transcription factor (TF) and long non-coding RNA (lncRNA) regulation for intersected genes. (a) network formed by six key transcription factors and their target genes. (b) possible long non-coding RNA (lncRNA) regulation in OA. CYTOR acts as competing endogenous RNA (ceRNA) to positively affect NRP1 expression by sponging with miR-206

Among these genes, we also identified 4 upregulated TFs – MAFB, HMGB3, GLI3, and SOX11 and 10 downregulated TFs – NR1D1, HMGB2, ZNF331, ZNF395, CEBPD, KLF11, ELF3, DDIT3, KLF10, and CEBPB. Compared to the previous studies (Fisch et al., 2018; Karlsson et al., 2010; Soul et al., 2018), 13 of these 14 TFs were overlapped and were in concordance with the direction of change (Supplementary Table S3). We also identified five upregulated lncRNAs – FZD10-AS1, PART1, ISM1-AS1, SILC1, and CYTOR and three downregulated lncRNAs – ILF3-DT, HG1, and S6-AS1. Compared to previous studies (Ajekigbe et al., 2019; Chen and Chen, 2020), four of these eight lncRNAs were overlapped and were in concordance with the direction of change (Supplementary Table S4). We obtained 38 significant key transcriptional regulators (Supplementary Table S2) for the 271 genes, of which TWIST2, MYBL2, RELA, JUN, KLF4, and PTTG1 were DEGs. In the network formed by the six key TFs and their targets, RELA regulated the most genes, and TWIST2 was the most significant (Figure 5a). We simultaneously found CYTOR and NRP1 within these genes. It was found in the LncTarD database that CYTOR functioned as a competing endogenous RNA (ceRNA) to positively regulate NRP1 expression by sponging with miRNA-206, which could positively affect epithelial to mesenchymal transition, cancer progression, and cell growth (Figure 5b).

## 4. Discussion

Knee OA is a disease characterized by degenerative changes in cartilage. Due to the imbalance between the breakdown and repair of cartilage, knee replacement surgery will be eventually required if there is an excess of cartilage erosion. Therefore, it is necessary to understand the molecular mechanism of pathological changes in knee cartilage in OA. The bulk RNA-seq dataset we investigated here provided upregulated or downregulated genes in the osteoarthritic cartilage compared to normal cartilage and gene modules most positively or negatively relevant to OA trait. The pseudotime analysis of scRNA-seq suggested genes changing expression with pathological stages of osteoarthritic cartilage. To identify genes that might have an important role in the pathology of OA, we intersected the genes suggested by the analysis of the bulk RNA-seq dataset with the genes suggested by the pseudotime analysis of scRNA-seq dataset. We found 271 shared genes, 183 of which were upregulated in osteoarthritic cartilage compared to normal cartilage and 88 of which were downregulated, according to the results of the bulk RNA-seq and scRNA-seq analysis. Of the 271 shared genes, we identified 14 TFs and 8 lncRNAs, their expression pattern was in high accordance with previous studies [6,10,11,23,24]. We also obtained 6 key transcriptional regulators for the 271 genes. Potential network formed by these six key transcriptional regulators was shown. Potential lncRNA regulation between lncRNA CYTOR and gene NRP1 was also suggested.

The pseudotime model we constructed approximately arranged the chondrocytes by the pathological stage of cartilage lesion. The chondrocytes from normal-looking cartilage were designed to be placed at the start of pseudotime and the chondrocytes from the most severely damaged cartilage were designed to be placed at the end of pseudotime. Many of the upregulated or downregulated genes of bulk-seq were upregulated at the end or start of pseudotime. The start of pseudotime could represent the normal or early-stage OA conditions, and the end of pseudotime could represent the end-stage of OA. The genes upregulated at the end of pseudotime might be biomarkers that reflect OA progression. As fibronectin 1 (FN1) was upregulated at the end of pseudotime, this might indicate that the secreted protein FN 1 could be a candidate biomarker for representing OA progression. Peffers et al. [25] also identified increased FN staining in OA cartilage compared to old cartilage.

The pathways enriched by the genes in the intersection seemed to be a subset of the pathways enriched by the DEGs or the genes in gene modules most relevant to OA, and indicated some promising pathways for the treatment of OA. HIF-1 signaling pathways were enriched by the downregulated genes at the intersection. HIF-1α is thought to protect articular cartilage by supporting metabolic adaptation to hypoxic environments and its expression was verified to increase with severity of OA [26]. Recent study has shown that the mitophagy mediated by HIF-1α could alleviate OA (Hu et al., 2020), which indicated HIF-1α a promising target for OA treatment.

In the transcriptional regulatory network formed by the genes at the intersection, we found that transcriptional PTTG1 and its downstream target gene S100A4, LGALS1 were both upregulated in osteoarthritic cartilage. There is a chance that the activated expression of S100A4 and LGALS1 is caused by the activated expression of PTTG1. S100A4 and LGALS1 both could promote inflammation and elicit catabolic signaling pathways [27,28]. Inhibiting the expression of PTTG1 might be beneficial to protect the knee cartilage in OA.

In our study, we found that NRP1 co-expressed with CYTOR as upregulated genes in osteoarthritic cartilage. It was reported that CYTOR positively regulated NRP1 expression by sponging with miRNA-206 in colorectal cancer [29], and miRNA-206 was significantly increased in human OA chondrocytes [30]. There is a possibility that the same lncRNA regulation exists in OA as well. As a co-receptor for both vascular endothelial growth factor (VEGF) and semaphoring, NRP1 can induce vasculogenesis and angiogenesis [31], which contribute to structural damage of cartilage and pain in OA. If so, blockade of this regulation might relieve pain and ameliorate the disease progression of OA.

There were some intrinsic drawbacks to our study. When analyzing the phenotypic characteristics of the bulk RNA-seq dataset, it was found that the age difference between the normal group and the OA group was large. The average age of the normal group was 36.6, while the average age of the OA group was 66.2. Whereas aging is a major risk factor for OA, therapeutic targets for age-related changes in chondrocytes might benefit the treatment of OA. The FOXO signaling pathway enriched by downregulated genes in the intersection was an age-related pathway. With the joint aging, the expression of FOXO1 and FOXO3 was reduced in the superficial zone of cartilage and the FOXO protein was deactivated in abnormal chondrocytes of OA cartilage [32]. It has been reported that the overexpression of FOXO1 protected the chondrocytes from OA damage [33]. Although there is no significant difference in the sex ratio between the normal group and the OA group, there is still a certain difference. Women accounted for 27.78% of the total in the normal group, while women accounted for 60% of total in the OA group. This research is only a bioinformatics study, and a large number of experimental studies are still needed to validate the proposed regulations and pathways here.

## 5. Conclusion

In summary, we obtained a series of key genes and revealed their possible expression patterns during OA progression by combining the analysis results of bulk RNA-seq and scRNA-seq. The TFs and lncRNAs were identified and key transcriptional regulators were explored. We also discovered potential regulation by lncRNA between CYTOR and NRP1, which could be linked to vascularization of the cartilage and involved with arthritic knee pain in OA. Further studies on the gene expression pattern and molecular regulation in OA chondrocytes are required to validate our findings.

## Supplementary Material

Supplemental Material

## Code availability:

The R language codes we used in this study have been uploaded to Github: https://github.com/XiaoyuLi-smu/GSE114007 and https://github.com/XiaoyuLi-smu/GSE104782.

## Highlights

1. Genes suggested by bulk and single-cell RNA sequencing analysis of knee osteoarthritis chondrocytes were explored.
2. A transcriptional regulatory network was constructed based on six key transcriptional regulators.
3. The lnc RNA regulation between CYTOR and NRP1 might exist in the osteoarthritic cartilage of knee joint.
