# Supplementary material for: Combining bulk and single-cell RNA-sequencing data to reveal gene expression pattern of chondrocytes in the osteoarthritic knee
Source: Bioengineered. 2021 Mar 22;12(1):997–1007. doi: 10.1080/21655979.2021.1903207 (PMC8806218; doi:10.1080/21655979.2021.1903207)
Supplement: Supplemental Material [file KBIE_A_1903207_SM8038.docx]

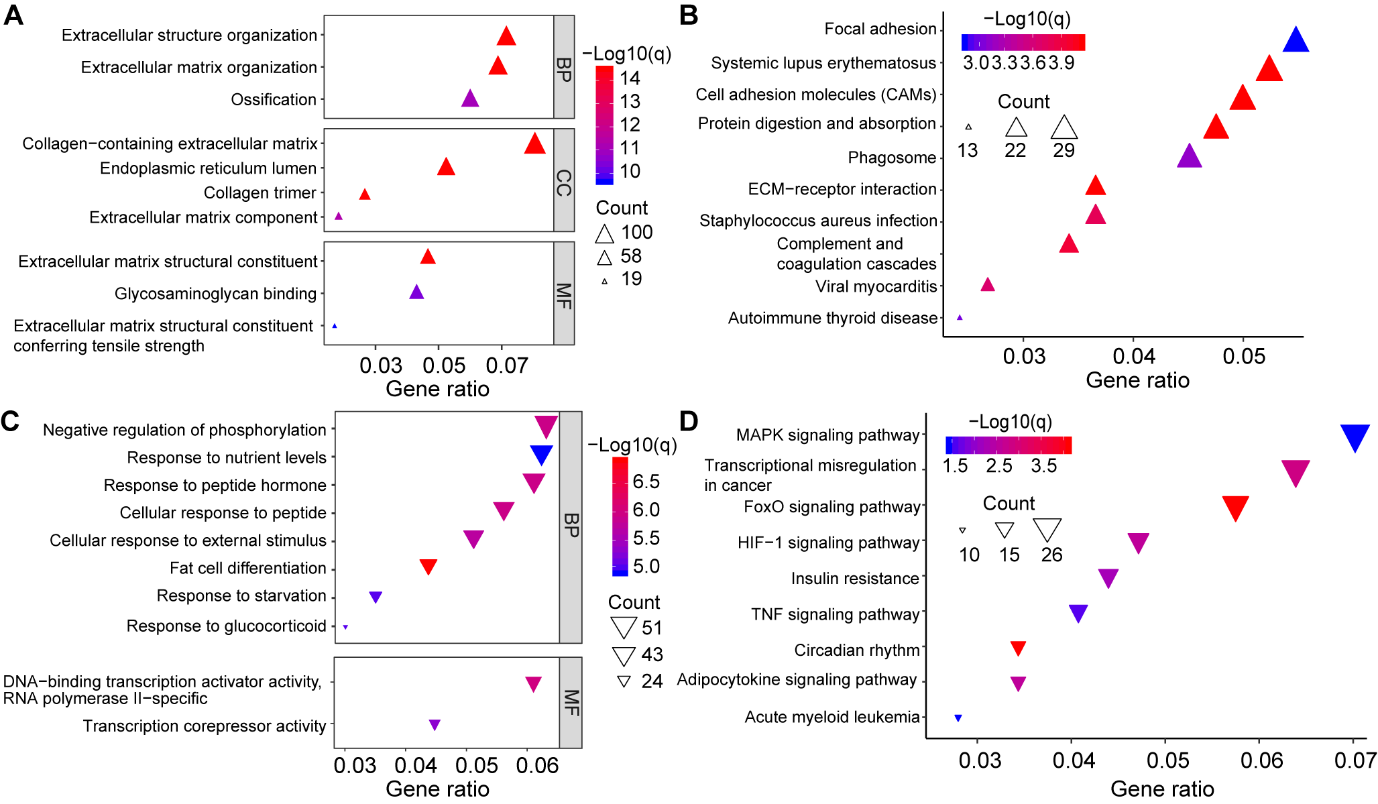


SUPPLEMENTARY FIG. S1. Gene Ontology (GO) and Kyoto Encyclopedia of Genes and Genomes (KEGG) pathways enriched by upregulated or downregulated differentially expressed genes (DEGs) in osteoarthritic chondrocytes. (A) Top 10 upregulated GO pathways ranked by q value. (B) Top 10 upregulated KEGG pathways ranked by q value. (C) Top 10 downregulated GO pathways ranked by q value. (D) Top 10 downregulated KEGG pathways ranked by q value. BP: biological process; CC: cellular component; MF: molecular function.


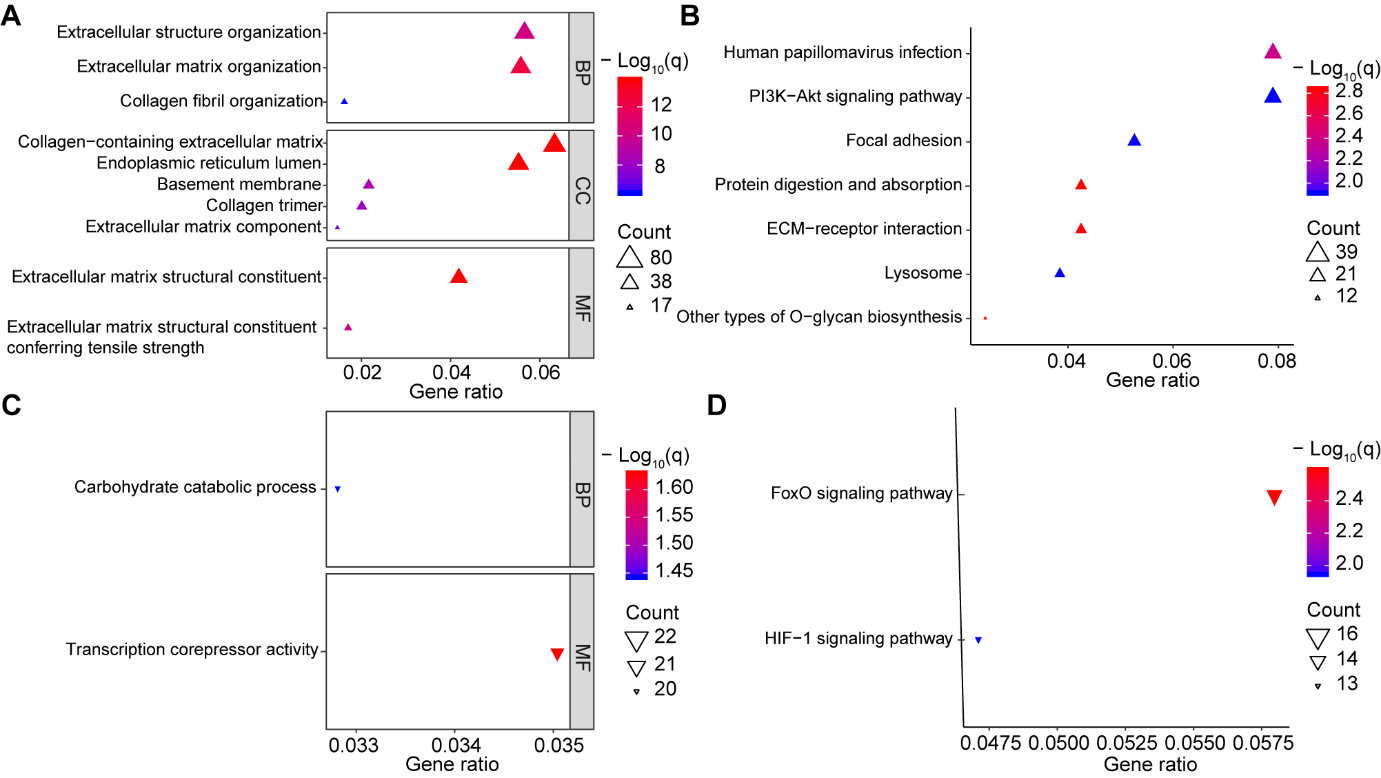


SUPPLEMENTARY FIG. S2. Gene Ontology (GO) and Kyoto Encyclopedia of Genes and Genomes (KEGG) pathways enriched by the genes of module having the strongest positive (A-B) and negative (C-D) correlation with osteoarthritis. BP: biological process; CC: cellular component; MF: molecular function.


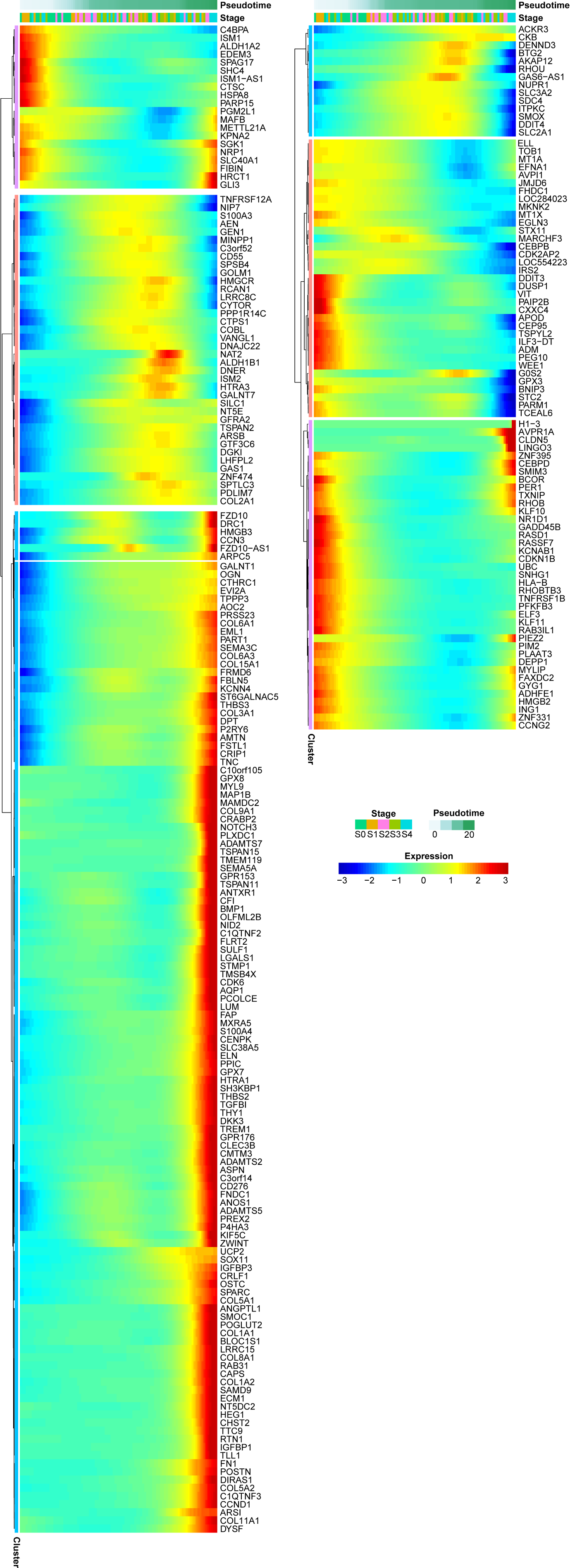


SUPPLEMENTARY FIG. S3. Expression pattern of genes in the intersection. The left panel indicated the expression of upregulated genes. The right panel indicated the expression of downregulated genes.

SUPPLEMENTARY TABLE S1. GENES OF INTERESTS

| *Type* | *Total* | *Gene* |
| --- | --- | --- |
| Upregulated | 183 | FSTL1 AEN RTN1 DIRAS1 CAPS LHFPL2 GEN1 COL1A1 C1QTNF3 ISM1 FNDC1 METTL21A CMTM3 PREX2 RCAN1 NT5E C1QTNF2 SPTLC3 KIF5C FZD10-AS1 UCP2 AMTN ASPN CD276 OGN MINPP1 HTRA1 ZNF474 GAS1 CRIP1 SULF1 MYL9 C10orf105 CENPK IGFBP3 C4BPA COL11A1 TREM1 COL1A2 ADAMTS7 ST6GALNAC5 PDLIM7 CD55 EML1 PART1 SLC40A1 ALDH1A2 ARPC5 FRMD6 ELN ARSB GPR176 CRLF1 LRRC15 TNFRSF12A COL3A1 DPT SPAG17 TSPAN15 OSTC POGLUT2 DGKI ANOS1 IGFBP1 ISM2 PRSS23 FLRT2 AQP1 COBL VANGL1 COL6A3 SHC4 GTF3C6 PPP1R14C COL6A1 SAMD9 SPARC COL5A1 TMEM119 CTHRC1 MAMDC2 ADAMTS5 NOTCH3 PLXDC1 FBLN5 FIBIN SLC38A5 PCOLCE GPR153 KPNA2 DYSF EVI2A P4HA3 C3orf52 CLEC3B HSPA8 ADAMTS2 STMP1 FN1 PGM2L1 S100A3 SGK1 COL5A2 THY1 ANTXR1 HRCT1 CCND1 TPPP3 MXRA5 ZWINT CHST2 FZD10 TSPAN2 GFRA2 COL8A1 ANGPTL1 HMGCR COL9A1 LUM PARP15 ISM1-AS1 CRABP2 DKK3 POSTN NAT2 CTPS1 BMP1 NT5DC2 NID2 GPX8 MAFB SPSB4 PPIC SILC1 GALNT1 EDEM3 AOC2 C3orf14 TSPAN11 HMGB3 GOLM1 NRP1 GPX7 DNAJC22 LRRC8C LGALS1 COL15A1 THBS3 ECM1 CDK6 SH3KBP1 ALDH1B1 HTRA3 GALNT7 GLI3 RAB31 HEG1 BLOC1S1 ARSI NIP7 TNC FAP DRC1 S100A4 P2RY6 CFI COL2A1 CYTOR THBS2 SMOC1 OLFML2B MAP1B SOX11 CCN3 SEMA5A TLL1 DNER TMSB4X TTC9 TGFBI KCNN4 SEMA3C CTSC |
| Downregulated | 83 | BTG2 FHDC1 ILF3-DT ELL GADD45B ADHFE1 SLC3A2 FAXDC2 PER1 NR1D1 ACKR3 CKB CDK2AP2 NUPR1 H1-3 TSPYL2 EFNA1 DUSP1 G0S2 MT1X LOC284023 TXNIP HMGB2 DDIT4 CLDN5 PLAAT3 TNFRSF1B RASD1 HLA-B EGLN3 GPX3 STC2 PEG10 TOB1 PARM1 UBC LOC554223 ZNF331 ZNF395 VIT PIEZO2 CEBPD TCEAL6 IRS2 KCNAB1 SLC2A1 WEE1 LINGO3 MKNK2 RHOU AKAP12 APOD ADM SNHG1 GAS6-AS1 MT1A GYG1 DEPP1 PIM2 RASSF7 KLF11 SMIM3 CDKN1B ELF3 CCNG2 BNIP3 SMOX SDC4 AVPI1 STX11 PFKFB3 BCOR CEP95 MYLIP JMJD6 DDIT3 DENND3 RHOB AVPR1A RAB3IL1 PAIP2B MARCHF3 RHOBTB3 CXXC4 ITPKC KLF10 ING1 CEBPB |

SUPPLEMENTARY TABLE S2. KEY TRANSCRIPTIONAL REGULATORS FOR THE GENES IN THE INTERSECTION

| *Key TF* | *Description* | *Number of overlapped genes* | *P value* | *Q value* | *List of overlapped genes* |
| --- | --- | --- | --- | --- | --- |
| ATF4 | activating transcription factor 4 (tax-responsive enhancer element B67) | 5 | 9.19E-05 | 3.91E-03 | DDIT3, NUPR1, CEBPB, DDIT4, IGFBP1 |
| VHL | von Hippel-Lindau tumor suppressor, E3 ubiquitin protein ligase | 4 | 1.23E-04 | 3.91E-03 | CCND1, SPARC, CDKN1B, KLF10 |
| HOXB7 | homeobox B7 | 3 | 2.57E-04 | 3.91E-03 | THBS2, CDKN1B, CCND1 |
| FOXO1 | forkhead box O1 | 4 | 2.59E-04 | 3.91E-03 | TXNIP, IRS2, IGFBP1, CDKN1B |
| PGR | progesterone receptor | 4 | 3.05E-04 | 3.91E-03 | DUSP1, CCND1, IRS2, IGFBP1 |
| CIITA | class II, major histocompatibility complex, transactivator | 4 | 7.14E-04 | 7.49E-03 | COL1A1, S100A4, COL1A2, HLA-B |
| HIF1A | hypoxia inducible factor 1, alpha subunit (basic helix-loop-helix transcription factor) | 6 | 8.19E-04 | 7.49E-03 | CCND1, NT5E, PFKFB3, BNIP3, EGLN3, SLC2A1 |
| STAT6 | signal transducer and activator of transcription 6, interleukin-4 induced | 4 | 1.27E-03 | 9.91E-03 | COL1A2, EGLN3, COL1A1, DUSP1 |
| TP53 | tumor protein p53 | 8 | 1.57E-03 | 9.91E-03 | ANTXR1, ING1, SLC2A1, CDKN1B, DUSP1, CCND1, BTG2, IGFBP3 |
| FHL2 | four and a half LIM domains 2 | 2 | 1.70E-03 | 9.91E-03 | CDKN1B, CCND1 |
| KDM4B | lysine (K)-specific demethylase 4B | 2 | 1.70E-03 | 9.91E-03 | WEE1, CCND1 |
| HDAC1 | histone deacetylase 1 | 5 | 2.50E-03 | 1.25E-02 | IGFBP3, TXNIP, CCND1, RHOB, COL1A2 |
| CEBPZ | CCAAT/enhancer binding protein (C/EBP), zeta | 2 | 2.53E-03 | 1.25E-02 | COL1A2, COL11A1 |
| NCOR1 | nuclear receptor corepressor 1 | 2 | 3.51E-03 | 1.58E-02 | IGFBP3, G0S2 |
| SIRT1 | sirtuin 1 | 4 | 3.70E-03 | 1.58E-02 | PER1, CCND1, COL1A2, ADAMTS5 |
| SP3 | Sp3 transcription factor | 6 | 3.95E-03 | 1.58E-02 | IGFBP3, IGFBP1, UBC, COL1A1, CCND1, COL2A1 |
| TWIST2 | twist basic helix-loop-helix transcription factor 2 | 3 | 4.26E-03 | 1.60E-02 | POSTN, FN1, CTPS1 |
| MYBL2 | v-myb myeloblastosis viral oncogene homolog (avian)-like 2 | 2 | 4.64E-03 | 1.65E-02 | CCND1, COL1A1 |
| EP300 | E1A binding protein p300 | 4 | 6.44E-03 | 2.17E-02 | CCND1, S100A4, IGFBP3, COL1A2 |
| RELA | v-rel reticuloendotheliosis viral oncogene homolog A (avian) | 10 | 7.01E-03 | 2.24E-02 | COL1A2, ELF3, BTG2, TNC, CDK6, CCND1, COL1A1, CFI, COL2A1, FN1 |
| TFAP2C | transcription factor AP-2 gamma (activating enhancer binding protein 2 gamma) | 2 | 7.34E-03 | 2.24E-02 | CRABP2, ECM1 |
| AR | androgen receptor | 5 | 7.91E-03 | 2.30E-02 | CDK6, FN1, WEE1, IGFBP3, BTG2 |
| ATF2 | activating transcription factor 2 | 3 | 8.58E-03 | 2.37E-02 | FN1, CCND1, DUSP1 |
| MECP2 | methyl CpG binding protein 2 (Rett syndrome) | 2 | 8.89E-03 | 2.37E-02 | IGFBP3, CCND1 |
| STAT3 | signal transducer and activator of transcription 3 (acute-phase response factor) | 6 | 1.17E-02 | 2.99E-02 | KLF11, UCP2, AKAP12, CCND1, CDKN1B, DDIT3 |
| JUN | jun proto-oncogene | 6 | 1.45E-02 | 3.38E-02 | CCND1, DDIT3, TNC, TNFRSF1B, ELN, RHOB |
| TFAP2A | transcription factor AP-2 alpha (activating enhancer binding protein 2 alpha) | 4 | 1.46E-02 | 3.38E-02 | CRABP2, ECM1, ADM, COL1A1 |
| PPARA | peroxisome proliferator-activated receptor alpha | 3 | 1.48E-02 | 3.38E-02 | TXNIP, G0S2, IGFBP1 |
| KLF4 | Kruppel-like factor 4 (gut) | 3 | 1.58E-02 | 3.38E-02 | CDKN1B, HSPA8, CCND1 |
| ING4 | inhibitor of growth family, member 4 | 2 | 1.64E-02 | 3.38E-02 | CDKN1B, CCND1 |
| PTTG1 | pituitary tumor-transforming 1 | 2 | 1.64E-02 | 3.38E-02 | S100A4, LGALS1 |
| VDR | vitamin D (1, 25- dihydroxyvitamin D3) receptor | 3 | 1.80E-02 | 3.56E-02 | DDIT4, IGFBP3, CDKN1B |
| ESR1 | estrogen receptor 1 | 4 | 1.83E-02 | 3.56E-02 | BTG2, CDKN1B, CEBPB, CCND1 |
| NFKB1 | nuclear factor of kappa light polypeptide gene enhancer in B-cells 1 | 9 | 2.00E-02 | 3.76E-02 | TNC, COL2A1, COL1A2, ELF3, FN1, CFI, BTG2, CCND1, COL1A1 |
| MYCN | v-myc myelocytomatosis viral related oncogene, neuroblastoma derived (avian) | 3 | 2.17E-02 | 3.96E-02 | DKK3, CDKN1B, HLA-B |
| SP1 | Sp1 transcription factor | 12 | 2.38E-02 | 4.23E-02 | CDKN1B, NT5E, TNC, UBC, COL2A1, IGFBP3, COL1A1, IRS2, CDK6, SLC3A2, IGFBP1, CCND1 |
| KLF6 | Kruppel-like factor 6 | 2 | 2.84E-02 | 4.78E-02 | CCND1, TXNIP |
| NFYA | nuclear transcription factor Y, alpha | 2 | 2.84E-02 | 4.78E-02 | DDIT3, CDKN1B |
| AHR | aryl hydrocarbon receptor | 2 | 3.39E-02 | 5.43E-02 | CCNG2, CCND1 |
| ATM | ataxia telangiectasia mutated | 2 | 3.39E-02 | 5.43E-02 | DUSP1, SLC2A1 |
| E2F4 | E2F transcription factor 4, p107/p130-binding | 2 | 3.68E-02 | 5.61E-02 | PEG10, CCND1 |
| NANOG | Nanog homeobox | 2 | 3.68E-02 | 5.61E-02 | CDK6, CCND1 |
| BRCA1 | breast cancer 1, early onset | 3 | 3.98E-02 | 5.80E-02 | CCND1, CDKN1B, DDIT3 |
| FLI1 | Friend leukemia virus integration 1 | 2 | 3.99E-02 | 5.80E-02 | COL1A2, IGFBP3 |
| HNF1A | HNF1 homeobox A | 2 | 4.61E-02 | 6.42E-02 | NT5E, IGFBP1 |
| SMAD4 | SMAD family member 4 | 2 | 4.61E-02 | 6.42E-02 | UCP2, TNC |
| NFIC | nuclear factor I/C (CCAAT-binding transcription factor) | 2 | 4.94E-02 | 6.73E-02 | COL1A1, IRS2 |
| LEF1 | lymphoid enhancer-binding factor 1 | 2 | 5.62E-02 | 7.49E-02 | NT5E, CCND1 |
| RUNX3 | runt-related transcription factor 3 | 2 | 5.97E-02 | 7.79E-02 | CCND1, ING1 |
| RB1 | retinoblastoma 1 | 2 | 6.32E-02 | 8.09E-02 | TGFBI, DDIT3 |
| REST | RE1-silencing transcription factor | 2 | 6.69E-02 | 8.39E-02 | NRP1, KCNN4 |
| TWIST1 | twist basic helix-loop-helix transcription factor 1 | 2 | 7.82E-02 | 9.63E-02 | CTPS1, FN1 |
| MYB | v-myb myeloblastosis viral oncogene homolog (avian) | 2 | 8.61E-02 | 1.03E-01 | COL1A1, COL1A2 |
| ETS1 | v-ets erythroblastosis virus E26 oncogene homolog 1 (avian) | 3 | 8.74E-02 | 1.03E-01 | IGFBP1, TNC, COL1A1 |
| NR3C1 | nuclear receptor subfamily 3, group C, member 1 (glucocorticoid receptor) | 2 | 9.01E-02 | 1.03E-01 | CCND1, CDK6 |
| POU2F1 | POU class 2 homeobox 1 | 2 | 9.01E-02 | 1.03E-01 | SDC4, CCND1 |
| EZH2 | enhancer of zeste homolog 2 (Drosophila) | 2 | 9.83E-02 | 1.10E-01 | IGFBP1, CCND1 |
| E2F1 | E2F transcription factor 1 | 4 | 1.03E-01 | 1.13E-01 | PEG10, CCND1, DUSP1, DDIT3 |
| EGR1 | early growth response 1 | 3 | 1.12E-01 | 1.21E-01 | FAP, FN1, CCND1 |
| MYC | v-myc myelocytomatosis viral oncogene homolog (avian) | 3 | 1.47E-01 | 1.57E-01 | HLA-B, CCND1, CDK6 |
| FOS | FBJ murine osteosarcoma viral oncogene homolog | 2 | 1.74E-01 | 1.83E-01 | CCND1, DDIT3 |
| CEBPB | CCAAT/enhancer binding protein (C/EBP), beta | 2 | 1.89E-01 | 1.95E-01 | ADM, DDIT3 |
| PPARG | peroxisome proliferator-activated receptor gamma | 2 | 2.18E-01 | 2.21E-01 | CCND1, TXNIP |
| YY1 | YY1 transcription factor | 2 | 3.40E-01 | 3.40E-01 | COL1A2, POSTN |

SUPPLEMENTARY TABLE S3. TRANSCRIPTION FACTORS IN THE INTERSECTION AND COMPARISON WITH PREVIOUS STUDIES

| *Gene name* | *Differentially expressed in our study* | *Differentially expressed in Fisch et al* | *Concordant with our study (Fisch et al)* | *Differentially expressed in Soul et al* | *Concordant with our study (Soul et al)* | *Differentially expressed in Karlsson et al* | *Concordant with our study (Karlsson et al)* |
| --- | --- | --- | --- | --- | --- | --- | --- |
| CEBPB | Downregulated | Downregulated | Yes | Downregulated | Yes | Downregulated | Yes |
| CEBPD | Downregulated |  |  | Downregulated | Yes | Downregulated | Yes |
| DDIT3 | Downregulated | Downregulated | Yes | Downregulated | Yes |  |  |
| ELF3 | Downregulated | Downregulated | Yes | Downregulated | Yes |  |  |
| GLI3 | Upregulated | Upregulated | Yes | Upregulated | Yes | Upregulated | Yes |
| HMGB2 | Downregulated | Downregulated | Yes | Downregulated | Yes | Downregulated | Yes |
| HMGB3 | Upregulated |  |  |  |  |  |  |
| KLF10 | Downregulated | Downregulated | Yes | Downregulated | Yes |  |  |
| KLF11 | Downregulated | Downregulated | Yes | Downregulated | Yes |  |  |
| MAFB | Upregulated | Upregulated | Yes |  |  | Upregulated | Yes |
| NR1D1 | Downregulated | Downregulated | Yes | Downregulated | Yes |  |  |
| SOX11 | Upregulated | Upregulated | Yes | Upregulated | Yes | Upregulated | Yes |
| ZNF331 | Downregulated | Downregulated | Yes |  |  |  |  |
| ZNF395 | Downregulated | Downregulated | Yes |  |  | Downregulated | Yes |

SUPPLEMENTARY TABLE S4. LONG NON-CODING RNAS IN THE INTERSECTION AND COMPARISON WITH PREVIOUS STUDIES

| *Gene name* | *Differentially expressed in our study* | *Differentially expressed in Chen et al* | *Concordant with our study (Chen et al)* | *Differentially expressed in Ajekigbe et al* | *Concordant with our study (Ajekigbe et al)* |
| --- | --- | --- | --- | --- | --- |
| CYTOR | Upregulated |  |  | Upregulated | Yes |
| FZD10-AS1 | Upregulated |  |  |  |  |
| GAS6-AS1 | Downregulated |  |  |  |  |
| ILF3-DT | Downregulated | Downregulated | Yes | Downregulated | Yes |
| ISM1-AS1 | Upregulated | Upregulated | Yes |  |  |
| PART1 | Upregulated | Upregulated | Yes |  |  |
| SILC1 | Upregulated |  |  |  |  |
| SNHG1 | Downregulated |  |  |  |  |
